# 2-Amino­terephthalic acid–4,4′-bipyridine (1/1)

**DOI:** 10.1107/S1600536811016448

**Published:** 2011-05-07

**Authors:** Wenzhi Xiao, Ruiting Xue, Yansheng Yin

**Affiliations:** a Department of Physics and Mathematics, Hunan Institute of Engineering, Xiangtan 411104, People’s Republic of China; b Institute of Material Science and Engineering, Ocean University of China, Qingdao, Shandong 266100, People’s Republic of China

## Abstract

The asymmetric unit of the title compound, C_10_H_8_N_2_·C_8_H_7_NO_4_, contains two half-mol­ecules, which constitute a 1:1 co-crystal. The 2-amino­terephthalic acid mol­ecule is situated on an inversion center being disordered between two orientations in a 1:1 ratio. In the 4,4′-bipyridine mol­ecule, which is situated on a twofold rotational axis, the two pyridine rings form a dihedral angle of 37.5 (1)°. In the crystal, mol­ecules are held together *via* inter­molecular N—H⋯O and O—H⋯N hydrogen bonds. The crystal packing exhibits π–π inter­actions between the aromatic rings with a centroid–centroid distance of 3.722 (3) Å.

## Related literature

For the crystal structures of polymeric coordination polymers with 2-amino­terephthalic acid linkers, see: Ma *et al.* (2005) ▶; Bauer *et al.* (2008 ▶).
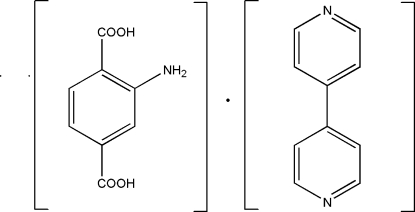


## Experimental

### 

#### Crystal data

C_10_H_8_N_2_·C_8_H_7_NO_4_
*M* *_r_* = 337.33
Monoclinic, 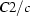

*a* = 16.9501 (18) Å
*b* = 11.1959 (13) Å
*c* = 9.251 (1) Å
β = 116.986 (2)°
*V* = 1564.4 (3) Å^3^
*Z* = 4
Mo *K*α radiation
μ = 0.10 mm^−1^
*T* = 293 K
0.33 × 0.19 × 0.11 mm

#### Data collection

Bruker SMART CCD area-detector diffractometer
Absorption correction: multi-scan (*SADABS*; Sheldrick, 1996 ▶) *T*_min_ = 0.967, *T*_max_ = 0.989
3865 measured reflections
1370 independent reflections
894 reflections with *I* > 2σ(*I*)
*R*_int_ = 0.075

#### Refinement

*R*[*F* ^2^ > 2σ(*F* ^2^)] = 0.067
*wR*(*F* ^2^) = 0.199
*S* = 1.00
1370 reflections
118 parameters
H-atom parameters constrained
Δρ_max_ = 0.32 e Å^−3^
Δρ_min_ = −0.29 e Å^−3^

### 

Data collection: *SMART* (Bruker, 2007 ▶); cell refinement: *SAINT* (Bruker, 2007 ▶); data reduction: *SAINT*; program(s) used to solve structure: *SHELXS97* (Sheldrick, 2008 ▶); program(s) used to refine structure: *SHELXL97* (Sheldrick, 2008 ▶); molecular graphics: *SHELXTL* (Sheldrick, 2008 ▶); software used to prepare material for publication: *SHELXTL*.

## Supplementary Material

Crystal structure: contains datablocks global, I. DOI: 10.1107/S1600536811016448/cv5085sup1.cif

Structure factors: contains datablocks I. DOI: 10.1107/S1600536811016448/cv5085Isup2.hkl

Supplementary material file. DOI: 10.1107/S1600536811016448/cv5085Isup3.cml

Additional supplementary materials:  crystallographic information; 3D view; checkCIF report

## Figures and Tables

**Table 1:** Hydrogen-bond geometry (Å, °)

*D*—H⋯*A*	*D*—H	H⋯*A*	*D*⋯*A*	*D*—H⋯*A*
N1—H1*A*⋯O2^i^	0.86	2.08	2.931 (5)	172
N1—H1*B*⋯O2^ii^	0.86	1.95	2.619 (5)	133
O1—H1⋯N2^iii^	0.82	1.82	2.635 (3)	170

Symmetry codes: (i) 
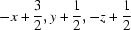
; (ii) 
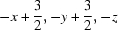
; (iii) 
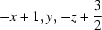
.

**Fig. 1.**
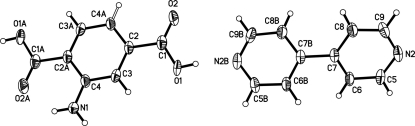
The structure of the title compound, showing 30% probability displacement ellipsoids and the atom-numbering scheme. (symmetry code A: x + 3/2, -y + 3/2, -z and B: -x + 1, y, -z + 3/2). For the disordered molecule of 2-aminoterephthalic acid, only one orientation is shown.

## supplementary crystallographic information

### Comment

Bipyridine is a well known molecule often used as a linker in polymeric coordination compounds. 2-Aminoterephthalic acid is also sometimes used as a linker in polymeric compounds (Ma *et al.*, 2005; Bauer *et al.*, 2008). The title compound (I) is a 1:1 cocrystal of the aforementioned linkers. Herewith we present its crystal structure.

In (I) (Fig. 1), the 2-aminoterephthalic acid molecule is situated on an inversion center, therefore, it is disordered (namely, the amino group is disordered between two positions). The carboxyl groups are twisted from the benzene ring plane at 11.4 (1)°. The 4,4'-bipyridine molecule is situated on a twofold rotational axis, and two pyridine rings form a dihedral angle of 37.5 (1)°.

In the crystal structure, the molecules are held together *via* intermolecular N—H···O and O—H···N hydrogen bonds. The crystal packing exhibits π—π interactions between the aromatic rings with the centroid-to-centroid distance of 3.722 (3) Å.

### Experimental

2-Diaminoterephthalic acid(10 mmol) and 4,4'-bipyridine(10 mmol) were dissolved to methanol (10 ml) and then hydrochloric acid(5 ml) was added. A few minutes later, an methanol solution (10 ml) of tin tetrachloride (5 mmol) was added with stirring. The reaction mixture was stirred for 4 h. The solution was held at room temperature for about two weeks, whereupon yellow crystals of the title compound, which were beyond expectations, were obtained.

### Refinement

All H-atoms were positioned geometrically and refined using a riding model, with C—H = 0.93 Å, N—H = 0.86 Å, O—H =0.82 Å and *U*_iso_(H) =1.2*U*_eq_(C), *U*_iso_(H) =1.2*U*_eq_(N), *U*_iso_(H) =1.5*U*_eq_(O).

### Crystal data

**Table:**

C_10_H_8_N_2_·C_8_H_7_NO_4_	*F*(000) = 704
*M**_r_* = 337.33	*D*_x_ = 1.432 Mg m^−^^3^
Monoclinic, *C*2/*c*	Mo *K*α radiation, λ = 0.71073 Å
*a* = 16.9501 (18) Å	Cell parameters from 1178 reflections
*b* = 11.1959 (13) Å	θ = 2.3–26.0°
*c* = 9.251 (1) Å	µ = 0.10 mm^−^^1^
β = 116.986 (2)°	*T* = 293 K
*V* = 1564.4 (3) Å^3^	Block, yellow
*Z* = 4	0.33 × 0.19 × 0.11 mm

### Data collection

**Table:**

Bruker SMART CCD area-detector diffractometer	1370 independent reflections
Radiation source: fine-focus sealed tube	894 reflections with *I* > 2σ(*I*)
graphite	*R*_int_ = 0.075
φ and ω scans	θ_max_ = 25.0°, θ_min_ = 2.3°
Absorption correction: multi-scan (*SADABS*; Sheldrick, 1996)	*h* = −20→12
*T*_min_ = 0.967, *T*_max_ = 0.989	*k* = −11→13
3865 measured reflections	*l* = −10→10

### Refinement

**Table:**

Refinement on *F*^2^	Primary atom site location: structure-invariant direct methods
Least-squares matrix: full	Secondary atom site location: difference Fourier map
*R*[*F*^2^ > 2σ(*F*^2^)] = 0.067	Hydrogen site location: inferred from neighbouring sites
*wR*(*F*^2^) = 0.199	H-atom parameters constrained
*S* = 1.00	*w* = 1/[σ^2^(*F*_o_^2^) + (0.1295*P*)^2^] where *P* = (*F*_o_^2^ + 2*F*_c_^2^)/3
1370 reflections	(Δ/σ)_max_ < 0.001
118 parameters	Δρ_max_ = 0.32 e Å^−^^3^
0 restraints	Δρ_min_ = −0.29 e Å^−^^3^

### Special details

**Table:**

Geometry. All e.s.d.'s (except the e.s.d. in the dihedral angle between two l.s. planes) are estimated using the full covariance matrix. The cell e.s.d.'s are taken into account individually in the estimation of e.s.d.'s in distances, angles and torsion angles; correlations between e.s.d.'s in cell parameters are only used when they are defined by crystal symmetry. An approximate (isotropic) treatment of cell e.s.d.'s is used for estimating e.s.d.'s involving l.s. planes.
Refinement. Refinement of *F*^2^ against ALL reflections. The weighted *R*-factor *wR* and goodness of fit *S* are based on *F*^2^, conventional *R*-factors *R* are based on *F*, with *F* set to zero for negative *F*^2^. The threshold expression of *F*^2^ > σ(*F*^2^) is used only for calculating *R*-factors(gt) *etc*. and is not relevant to the choice of reflections for refinement. *R*-factors based on *F*^2^ are statistically about twice as large as those based on *F*, and *R*- factors based on ALL data will be even larger.

### Fractional atomic coordinates and isotropic or equivalent isotropic displacement parameters (Å^2^)

**Table:**

	*x*	*y*	*z*	*U*_iso_*/*U*_eq_	Occ. (<1)
N1	0.8270 (3)	0.9576 (4)	−0.0096 (6)	0.0607 (13)	0.50
H1A	0.8317	1.0134	0.0577	0.073*	0.50
H1B	0.8478	0.9678	−0.0779	0.073*	0.50
N2	0.39951 (15)	0.8071 (2)	1.0092 (3)	0.0600 (7)	
O1	0.67530 (15)	0.82272 (19)	0.2964 (3)	0.0742 (8)	
H1	0.6550	0.8097	0.3602	0.111*	
O2	0.65519 (15)	0.62839 (19)	0.2565 (3)	0.0711 (7)	
C1	0.68006 (18)	0.7229 (3)	0.2283 (3)	0.0520 (8)	
C2	0.71594 (15)	0.7367 (2)	0.1106 (3)	0.0436 (7)	
C3	0.75521 (17)	0.8419 (2)	0.1014 (3)	0.0459 (7)	
H3	0.7595	0.9041	0.1713	0.055*	
C4	0.78845 (17)	0.8581 (2)	−0.0081 (3)	0.0462 (7)	
H4	0.8134	0.9307	−0.0140	0.055*	0.50
C5	0.39774 (19)	0.9000 (3)	0.9217 (3)	0.0594 (8)	
H5	0.3689	0.9685	0.9295	0.071*	
C6	0.43583 (18)	0.9020 (2)	0.8196 (3)	0.0529 (8)	
H6	0.4328	0.9707	0.7606	0.063*	
C7	0.47874 (16)	0.8020 (2)	0.8042 (3)	0.0451 (7)	
C8	0.47940 (19)	0.7037 (3)	0.8939 (3)	0.0564 (8)	
H8	0.5062	0.6331	0.8861	0.068*	
C9	0.4404 (2)	0.7104 (3)	0.9947 (4)	0.0646 (9)	
H9	0.4427	0.6436	1.0563	0.077*	

### Atomic displacement parameters (Å^2^)

**Table:**

	*U*^11^	*U*^22^	*U*^33^	*U*^12^	*U*^13^	*U*^23^
N1	0.094 (4)	0.048 (2)	0.071 (3)	−0.013 (2)	0.064 (3)	−0.013 (2)
N2	0.0592 (15)	0.0888 (17)	0.0500 (14)	−0.0024 (13)	0.0404 (13)	0.0035 (12)
O1	0.1089 (18)	0.0821 (14)	0.0749 (15)	0.0006 (12)	0.0797 (15)	−0.0074 (11)
O2	0.0983 (17)	0.0765 (14)	0.0719 (15)	0.0073 (12)	0.0679 (14)	0.0144 (11)
C1	0.0551 (16)	0.0688 (19)	0.0402 (15)	0.0119 (14)	0.0287 (13)	0.0078 (13)
C2	0.0456 (14)	0.0573 (15)	0.0366 (14)	0.0069 (12)	0.0264 (12)	0.0046 (11)
C3	0.0504 (15)	0.0575 (15)	0.0381 (14)	0.0071 (12)	0.0273 (13)	−0.0029 (11)
C4	0.0520 (16)	0.0550 (15)	0.0440 (15)	0.0023 (12)	0.0324 (13)	0.0046 (11)
C5	0.0643 (18)	0.0808 (19)	0.0509 (18)	0.0050 (15)	0.0416 (16)	−0.0027 (14)
C6	0.0640 (17)	0.0658 (17)	0.0448 (15)	0.0036 (14)	0.0385 (14)	0.0031 (12)
C7	0.0429 (14)	0.0644 (16)	0.0342 (13)	−0.0031 (12)	0.0229 (12)	−0.0010 (11)
C8	0.0624 (17)	0.0661 (17)	0.0552 (17)	0.0057 (14)	0.0394 (15)	0.0089 (13)
C9	0.073 (2)	0.0802 (19)	0.0576 (18)	−0.0005 (16)	0.0443 (17)	0.0180 (15)

### Geometric parameters (Å, °)

**Table:**

N1—C4	1.295 (5)	C3—H3	0.9300
N1—H1A	0.8600	C4—C2^i^	1.403 (4)
N1—H1B	0.8600	C4—H4	0.9300
N2—C5	1.310 (3)	C5—C6	1.365 (3)
N2—C9	1.325 (4)	C5—H5	0.9300
O1—C1	1.304 (3)	C6—C7	1.378 (3)
O1—H1	0.8200	C6—H6	0.9300
O2—C1	1.210 (3)	C7—C8	1.376 (3)
C1—C2	1.476 (4)	C7—C7^ii^	1.477 (5)
C2—C3	1.374 (4)	C8—C9	1.368 (4)
C2—C4^i^	1.403 (4)	C8—H8	0.9300
C3—C4	1.376 (4)	C9—H9	0.9300
			
C4—N1—H1A	120.0	C3—C4—H4	120.5
C4—N1—H1B	120.0	C2^i^—C4—H4	120.5
H1A—N1—H1B	120.0	N2—C5—C6	123.8 (3)
C5—N2—C9	116.9 (2)	N2—C5—H5	118.1
C1—O1—H1	109.5	C6—C5—H5	118.1
O2—C1—O1	122.8 (3)	C5—C6—C7	119.7 (3)
O2—C1—C2	123.5 (3)	C5—C6—H6	120.1
O1—C1—C2	113.7 (3)	C7—C6—H6	120.1
C3—C2—C4^i^	119.0 (2)	C8—C7—C6	116.6 (2)
C3—C2—C1	120.6 (2)	C8—C7—C7^ii^	122.47 (18)
C4^i^—C2—C1	120.4 (2)	C6—C7—C7^ii^	120.88 (17)
C2—C3—C4	122.0 (2)	C9—C8—C7	119.5 (3)
C2—C3—H3	119.0	C9—C8—H8	120.2
C4—C3—H3	119.0	C7—C8—H8	120.2
N1—C4—C3	119.8 (3)	N2—C9—C8	123.4 (3)
N1—C4—C2^i^	121.1 (3)	N2—C9—H9	118.3
C3—C4—C2^i^	119.0 (2)	C8—C9—H9	118.3
N1—C4—H4	3.0		

Symmetry codes: (i) −*x*+3/2, −*y*+3/2, −*z*; (ii) −*x*+1, *y*, −*z*+3/2.

### Hydrogen-bond geometry (Å, °)

**Table:**

*D*—H···*A*	*D*—H	H···*A*	*D*···*A*	*D*—H···*A*
N1—H1A···O2^iii^	0.86	2.08	2.931 (5)	172
N1—H1B···O2^i^	0.86	1.95	2.619 (5)	133
O1—H1···N2^ii^	0.82	1.82	2.635 (3)	170

Symmetry codes: (iii) −*x*+3/2, *y*+1/2, −*z*+1/2; (i) −*x*+3/2, −*y*+3/2, −*z*; (ii) −*x*+1, *y*, −*z*+3/2.
